# Evaluation of Biological Properties and Beneficial Effects for a Sustainable and Conscious Exploitation of *Achatina fulica* Snails

**DOI:** 10.3390/biology14020190

**Published:** 2025-02-12

**Authors:** Andrea Alogna, Alessia Liboni, Roberta Rizzo

**Affiliations:** 1 Department of Environmental and Prevention Sciences, University of Ferrara, 44121 Ferrara, Italy; roberta.rizzo@unife.it; 2 Department of Chemical, Pharmaceutical and Agrarian Sciences, University of Ferrara, 44121 Ferrara, Italy; alessia.liboni@unife.it

**Keywords:** *Achatina fulica*, snail mucus, *Angiostrongylus cantonensis*, wound healing, economic profitability, antimicrobial efficacy

## Abstract

In recent decades, there has been significant worldwide interest in the emergence of a new invasive species known as *Achatina fulica*. This is due to its dangerous habits for the environment, its biological characteristics and the fact that it is the intermediate host of several nematode parasites, such as *Angiostrongylus cantonensis*. This land snail species is native to tropical African countries, but has been introduced, accidentally or deliberately, to other parts of the world to be used for different purposes and is now established in a large part of the tropics. Since the 1980s, hundreds of researchers have been interested in the beneficial properties of its mucus, ranging from the antimicrobial and anticancer properties to the use of its powdered shell as a biocatalyst. This literature review aims to objectively describe the positive and negative aspects associated with the spread of *A. fulica*, highlighting in particular the opportunities for the local populations deriving from a conscious exploitation of this mollusc.

## Simple Summary

The invasive giant African land snail, *Achatina fulica*, poses significant environmental and health risks due to its rapid spread and role as a host for harmful parasites. However, this species also presents interesting economic and scientific opportunities. Its mucus has shown potential for wound healing, antimicrobial, anti-inflammatory, and anticancer applications, while its calcium-rich shells can be used in biotechnology, construction, and even biofuel production. Snail farming offers a sustainable income source in tropical regions due to the snail’s high nutritional value. This review highlights both the threats and benefits associated with *A. fulica*, suggesting that proper management could transform an ecological challenge into an economic asset, fostering sustainable development and innovation across multiple industries.

## 1. Introduction

*Achatina fulica*, commonly known as the Giant African Land Snail, is a fascinating gastropod mollusc that has attracted attention for its distinctive features and widespread distribution [1]. Originating from East Africa, this snail species has established itself in different regions of the world, demonstrating its adaptability to different environments [2]. Its distinctive conical shell, characterized by distinct spiral patterns, is a hallmark of *A. fulica* [3]. It is perhaps best known for its remarkable size, often reaching up to eight inches in length [4]. As a simultaneous hermaphrodite, possessing both male and female reproductive organs, it engages in a unique and complex mating process [5]. Its prolific reproductive abilities have led to its invasive nature in certain ecosystems, where it can outcompete native species. *A. fulica* is of interest in medical research due to its mucus and haemolymph which contain compounds with potential therapeutic properties [6]. However, it is important to manage its presence carefully, as the snail is considered an agricultural pest and poses risks to local ecosystems [7].

The aim of this literature review is to provide not only an overview of the risks, but also a detailed examination of the opportunities related to its exploitation. This review aims to be as exhaustive as possible with regard to the state of the art and future prospects. This review article is the result of a rigorous search of all the available scientific evidence on *A. fulica.* The documents were collected using the main research engines for scientific literature and organized according to strict criteria as described below.

## 2. Materials and Methods

### 2.1. Search Strategy

**Figure 1:**
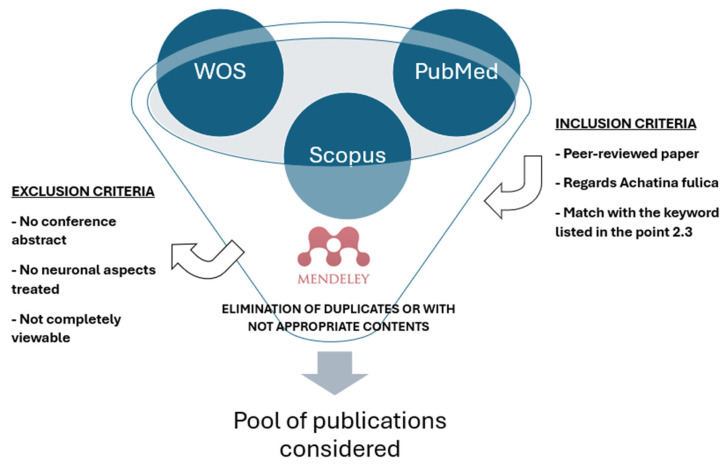
A flowchart illustrating the selection process for publications, highlighting inclusion and exclusion criteria from databases (WOS, PubMed, Scopus). The process involves filtering through Mendeley, eliminating duplicates and inappropriate content, ultimately leading to the final pool of considered publications.

We conducted a systematic search using electronic databases (PubMed, Scopus, Web of Sciences (WOS) and Science Open). The search was conducted until February 2024 using as main keywords/terms: “*A. fulica*” to restrict the research field on this argument and then adding other words, such as “pest control”, “nematode parasites”, “antimicrobial effect”, “wound healing”, “shell powder”, “*Angiostrongylus cantonensis*”, “cancer”, “reproduction cycle”, “distribution”, “dental diseases”, “neurons”, “evolution”, “Acharan sulphate”, “ecology”, “food studies” and “antiviral effect”. No date or language restrictions were applied (Figure 1).

### 2.2. Study Selection

We created a database of all the references collected and divided them up for discussion. Only peer-reviewed studies were included, and the selection of the studies was carried out by two independent reviewers with the aim of performing a title–abstract screening of all collected studies and then reviewing the full text of the selected articles. In cases where duplicate information was found from the same group of studies, the data were checked and combined but only considered as a single argument.

### 2.3. Inclusion Criteria

References providing information on *A. fulica* as the main subject of the study were selected. The publications were chosen based on positive and negative aspects related to *A. fulica* using specific keywords (“pest control,” “nematode parasites”, “antimicrobial effect”, “wound healing”, “shell powder”, “*Angiostrongylus cantonensis*”, “cancer”, “reproduction cycle”, “distribution”, “dental diseases”, “neurons”, “Evolution”, “Acharan sulphate”, “ecology”, “food studies” and “antiviral effect”) and Advanced Search Builder.

### 2.4. Exclusion Criteria

Studies that were not specifically related to the main topic treated, or that were only case reports, and commentaries were excluded. Moreover, publications without a DOI (e.g., conference abstracts and clinical trials) were excluded.

### 2.5. Data Extraction

**Figure 2:**
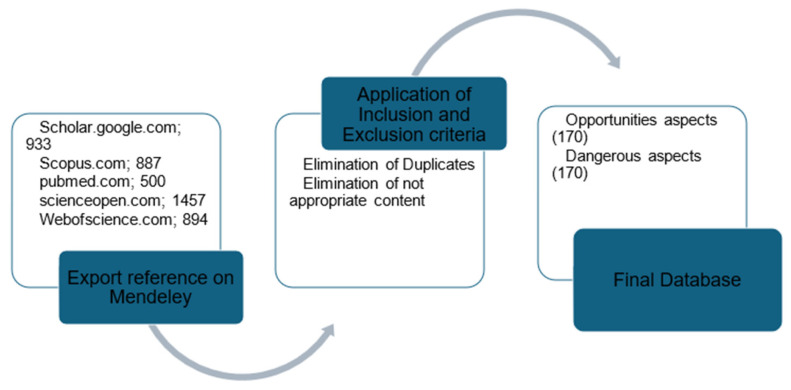
Data selection following eligibility criteria accordingly to the reviewed topic.

The data extraction process from the included studies was conducted by two independent reviewers, who considered key characteristics including publication year, author, type of study, country, sample size, and laboratory findings. In instances where the reviewers had made different selections, both were reported. Alternatively, the most accredited selection was reported based on other publications (Figure 2).

## 3. *A. Fulica*: Biology and Reproductive Cycle

*Achatina fulica* exhibits physiological properties typical of invertebrates. However, its status as an invasive species has spurred significant scientific interest and concern due to its unique traits and behaviors [8]. Its exceptional adaptability, high fecundity, and polyphagous diet have enabled *A. fulica* to successfully colonize diverse ecosystems worldwide, outcompeting native species and disrupting local biodiversity [9].

For these reasons, research has focused on exploring potential applications of this species, leveraging its beneficial characteristics.

Studies on circadian rhythms suggest that snail aggregation is influenced by age, genetic relationships and time of day [10]. Aggregation serves multiple purposes, including enhancing survival during unfavorable environmental conditions and facilitating reproduction by concentrating mating opportunities [11]. 

In addition, *A. fulica* exhibits unique dispersal patterns across different developmental stages, with juveniles dispersing over longer distances than adults [12,13,14]. 

Research on its optical system has demonstrated impressive regenerative capabilities in restoring phototransduction mechanisms, albeit with some limitations [15]. The accessory eye contains sensory cells resembling corneal cells, which have spectral sensitivities similar to those of the main eye, suggesting a role in monitoring light intensity [16].

The digestive system of *A. fulica* demonstrates specific hydrolase activities across a wide range of substrates, indicating its ability to digest various substances, including non-biological items and certain microorganisms [17,18]. In addition, its hepatopancreas plays a crucial role in digestion regulation and in responding to environmental stressors and treatments [19].

Investigations into cardiac activity have identified an undecapeptide that modulates ventricular beating though intricate neural signaling mechanisms, highlighting the complex interplay between neural signaling and biochemical modulation in cardi-ac function regulation [20,21].

Despite its simplicity, the nervous system of *A. fulica* has been extensively studied. It consists of several ganglia that contain giant neurons responsible for modulating the secretion of atrial glandular cells [22,23]. 

*A. fulica* also exhibits a remarkable development of the respiratory system, involving coordinated events from the formation of the lung primordia, through ectodermal invagination, to the establishment of functional respiratory organs that support gas exchange [24].

Moreover, these molluscs are characterized by a conical-shaped shell, with a length ranging from 4.8 to 102.5 mm [25]. Shell formation is a complex process involving glandular secretions and mesenchymal cell migration, with calcium deposition and enzymatic activity playing key roles [26].

Studies on the reproductive cycle have shown that *A. fulica* exhibits hermaphroditic characteristics, with reproductive activities influenced by environmental factors [6]. The sperm storage mechanisms suggest long-term viability, with hormonal regulation affecting oocyte production and mating behaviours, this in such ways influence its persistence in a new habitat, even in non-optimal conditions [5].

*A. fulica* produces snail slime through salivary epidermal glands in its foot [27]. This slime has potential benefits for human health, including moisturizing, lubricating, protective and repairing functions, as well as wound healing [28].

Various proteins have been identified within the mucus, such as proteoglycans, glycosaminoglycans (such as hyaluronic acid), glycoprotein enzymes, copper peptides, antimicrobial peptides and metal ions, which probably are at the base of its beneficial effects. Snail mucus also contains mucin, mitamycin-AF, allantoin, collagen, elastin, glycolic acid and achacin [29]. These substances interact synergistically, aiding in the snail’s movement and facilitating adhesion to substrates [30].

## 4. Threat for Ecosystems and Health

*Achatina fulica*, an omnivorous gastropod species, represents a significant threat to biodiversity and ecological equilibrium, in particular in tropical and subtropical regions [31]. Genomic insights into *Achatina fulica* have revealed several key adaptations that contribute to its ecological success as an invasive species. Studies comparing the genomes of *Achatina fulica* and other African land snails show that specific genetic traits, such as increased reproductive capacity and environmental tolerance, play a significant role in its ability to colonize diverse habitats [32,33].

Originating from East Africa this species rapidly has extended its habitat range far beyond its native environment due to both unintentional introductions and deliberate releases [34]. The spread of *A. fulica* has been influenced by various factors, including economic activities, religious practices, and historical events such as wars and trade. For example, religious traditions associated with certain African religions have contributed to its introduction to areas such as Cuba in 2014, where it is used in rituals [35,36,37,38]. In addition, commercial purposes, such as heliculture or snail consumption, have intentionally introduced this species in various regions of Brazil [39,40].

During and after the Second World War, Japanese merchants and soldiers further spread *A. fulica* throughout Southeast Asia, Taiwan and numerous Pacific Islands [41]. The invasive nature of these animals has been documented across continents, from Africa to the Caribbean [41], North and South America, Asia [23,42] and the Pacific Islands [43,44]. The presence of this gastropod in these areas has resulted in significant ecological disruption and economic losses, prompting efforts to control its spread. In certain areas, the introduction of *A. fulica* has resulted in the implementation of preventive measures and biological control methods, such as the introduction of predatory flatworms, too [45]. Even though the negative aspects resulting from their introduction have often outweighed the benefits.

**Figure 3:**
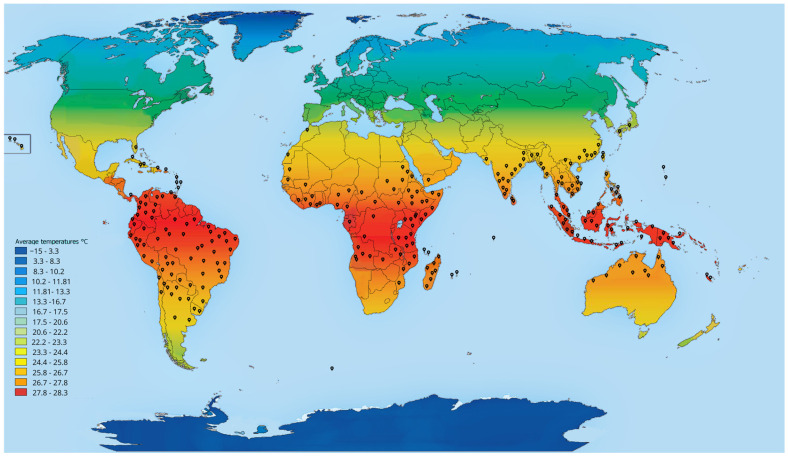
Current mean annual temperature (°C) estimates from WORLDCLIM data, with the global distribution of *Achatina fulica* (giant African snail) overlaid on average temperature gradients across continents. The map highlights the species’ adaptability to various climatic zones, with a preference for tropical and subtropical regions, based on observed and modeled data. Black dots indicate recorded occurrences of the species. For details on the distribution data, see Supplementary Table S1.

Overall, the widespread distribution (Figure 3) and ecological impact of *A. fulica* highlights the significance of comprehensive management strategies and intentional cooperation to mitigate its effects on global biodiversity and ecosystem health.

However, this snail species is well known even for the incredible number of nematodes and other parasites of which it is an intermediate host. The analysis of the 200 papers considered between 1965 and 2024 revealed that more than 12 nematode species were documented in association with *A. fulica* across 30 countries. Most studies reported associations between *A.fulica* and *Angiostrongylus cantonensis* [46,47,48,49,50,51,52]. Another parasite usually isolated from these animals was *Angiostrongylus malaysiensis* [53] and even if, to our knowledge, there is no evidence of natural infection, the potential transmission of *A. costaricensis* has also been experimentally demonstrated [54]. In addition to the presence of larvae of *A. cantonensis*, sporocysts of *Fasciola gigantica* and *Schistosoma mansoni* had been observed all together [55]. Three genera of Strongylida nematodes (Angiostrongylus, Aelurostrongylus and Strongyluris) were recorded as being associated with *A. fulica* specimens in the Valle del Cauca during 2013 and 2014 [56]. Nevertheless, *A. fulica* is not a threat only for humans but also for animal health. *Angiostrongylus vasorum* [57] and *Ancylostoma caninum* [58] have been reported too. *Aelurostrongylus abstrusus* larvae [59] have been recorded in the city of Guaratingueta, Sao Paulo State, and in the Amazon region demonstrating how diffused in the territory is this species and how dangerous could be it presence especially without any system of control [60].

## 5. Opportunities

*Achatina fulica* could represent a significant problem for environments and health, as previously described. Despite this, interesting opportunities can occur not only from the use of its mucus, but also from snail farming or why not from their shells. In the following paragraphs are summarized the main research evidence about beneficial effects of the snail mucus and alternative usages of shell and meat.

### 5.1. Gastronomic Use of the Animals and Alternative Use of Its Shell

To ensure safe consumption of mollusks, necessary precautions must be taken due to the risk of pathogens, as consuming uncooked or undercooked mollusks has led to documented infections [61]. Snail farming presents a potentially profitable venture requiring minimal investment in capital and land. Snails can be easily obtained from tropical regions and thrive in various environments like gardens, backyards, or cages, making them easy to farm [62].

*A. fulica* snail is native to sub-Saharan Africa, where it is commonly bred and consumed as a significant income source. Snail farming in Cameroon currently meets only about 25% of the national demand. Farm budgeting and profit analysis indicate a total production cost of approximately US$0.4 per kg, resulting in a five fold return on investment and a profit of US$2 per kg of snail sold [63]. Due to these economic benefits and growing international demand, *A. fulica* has been introduced to other tropical regions worldwide, including Brazil and Thailand; cultivation and processing of *A. fulica* could become a high-value activity in developing countries if its side effects are considered.

Snails are a cost-effective alternative to traditional meat, offering high protein content while being low in fat and cholesterol. Furthermore, by-products from snails can be used in the cosmetic and medicine industries, enhancing their economic value [64]. The bromatological analysis of the entire body showed a content of protein between 46% and 56%, a 2% carbohydrate content and just a 12% lipid dry weight content. The estimated content of calcium in both the soft body and shell consists of 20% and 25% of total ash, respectively. Instead, potassium is the next major element followed by magnesium and sodium in the soft body [64].

Another study highlighted these benefit too, sustaining that African giant snail (*Achatina fulica*) was nutritionally richer than the other snails considered in the study (e.g., *Helix pomatia*) in terms of protein and minerals, but on the other hand that in general they are also a reservoirs of pathogenic microorganisms which are of public health importance so great attention needs to be paid [65].

A recent Korean study underlined a presence of 40,4g of total amino acid on 44,2g of proteins on 100g dry matter, in particular Glutamic acid, and a significant presence of essential amino acids Leucine and Lysine. Mineral analysis of *A. fulica* revealed that it contains 2008.3 ± 0.15mg Calcium and 699.2 ± 0.03mg of Phosphorus on 100g dry matter [66]. 

Snails have been a part of the human diet since ancient times [67]. In some African countries, even the shells of *A. fulica* and other giant snail species are used for bleaching, brushing, abrasion, and other applications. A study in Nigeria tested them for antimicrobial effects but found none; however, nutritional analysis of 100g of shell powder showed high carbohydrate concentrations (83.54-92.76g) and low protein (0.16-0.38g). Fat content ranged between 0.42g and 0.82g, and ash between 2.14g and 9.45g. Calcium was the most abundant element (10.25-96.35mg/g), while Potassium was the least abundant (0.3-0.7mg/g) [68].

Notably, the systematic cultivation of *A. fulica* is not common in Europe or temperate regions due to its specific growth requirements and the absence of traditional consumption practices. Instead, they are often kept and sold in these area as pets in exotic animal fairs and are used in educational settings to help children develop caregiving attitudes and empathy [69].

Beyond their potential for food and other applications, *Achatina fulica* snail shells are being extensively investigated within a circular economic framework to generate added value for various sectors, including construction, biotechnology, and other environmentally sustainable applications.

Ecologically, the use of the Achatina shell by other Crustacea has been observed in various tropical areas of Brazil, aiding local diversity. Active ingredients from *A. fulica* shells, such as biogenic calcium carbonate (CaCO_3_) [70], and Chitosan derived through autoclave-(SSCA) or ultrasound-assisted (SSCU) deacetylation, have been successfully isolated and produced. Chitosan treatment has been shown to enhance the quality and shelf life of tomatoes and cucumbers, with SSCA showing better results than SSCU and the control [71]. These active ingredients can also be used in the feed and construction industries. In some countries, *A. fulica* serves as a source of duck fodder commonly used by local breeders to meet the protein needs of poultry farms. The shells, rich in calcium, have been proposed as precursors for different types of bioceramics [72,73]. Calcium oxide from *A. fulica* shells applied to synthesize biodiesel from waste cooking oil results in biodiesel with a density within the ASTM standard [74]. Research has also explored the use of catalysts prepared from *A. fulica* snail shells for biodiesel conversion, offering a low-cost and reusable option. Additionally, CaO prepared from abandoned *A. fulica* shells through calcination is a potential cost-effective and sustainable photocatalyst [75,76].

*Achatina fulica* snail shells are a promising source for nano-calcium carbonate (nano-CaCO_3_) production via mechanochemical synthesis [77,78]. This process typically involves dry milling the shells, followed by wet milling with different solvents to achieve nanoparticle sizes [79]. Resulting particle sizes range from 11.56 to 180.06 nm, varying based on the solvent used [77]. Ethanol has been shown to yield the smallest particle sizes. Nano-CaCO_3_ derived from *Achatina fulica* shells has demonstrated potential as a reinforcing agent in various materials. For example, incorporating these nanoparticles can improve the tensile strength and stiffness of epoxy nanocomposites. Nano-CaCO_3_ can also be used to produce bioplastic films [80]. These techniques offer a low-cost method for producing nano-CaCO_3_ with uniformity of crystal morphology and structure. The solvents used for milling do not adversely affect the chemical properties of the nano-CaCO_3_ [78].

Preparation of green synthesized silver nanoparticle (AgNPs)-doped hydroxyapatite (Ag/HA) utilizing *Curcuma longa* leaf extract and land snail (*A. fulica*) shell waste was performed. Antibacterial activity of the nanocomposite was evaluated against *E. coli, Staphylococcus aureus, Klebsiella pneumoniae, and Streptococcus pyogenes*. The results showed that the varied Ag content (1.0; 1.6; and 2.4% wt) influenced the nanoparticle distribution in the nanocomposite and enhanced the antibacterial feature [81]. 

Considering the high voracity and the wide range of vegetables and materials which *A. fulica* is able to consume, the study of its intestinal microorganisms, including lactic acid bacteria (LAB), and the research of potential bioactive molecules has always aroused great interest [17]. For example, an enzyme, called Phytase, was purified from *Aspergillus fumigatus* isolated from the gut of *A. fulica* able to catalyzes the stepwise hydrolysis of phytate into phosphorus and organophosphate compound and capable of reducing environmental pollution and metal chelating effect. This study showed that phytase may contribute significantly to the phytate degrading enzyme system in African giant snails and may serve a useful commercial purpose [82]. From the digestive tract of *A. fulica* have been also isolated five new potential probiotics that are resistant at pH values of 2.0, 2.5, 3.0, and 4.0 and bile salt concentrations of 0.2%, 0.3%, 0.5%, 1%, and 2% expanding the application fields of this species further [83]. Polystyrene biodegradation has been also demonstrated by gut microorganisms of *A. fulica* after its ingestion showing once again how unique is its digestive system and how potentially useful could be [84].

### 5.2. Snail Mucus Effects

Research into the potential biomedical applications of snail mucus has gained interest due to its therapeutic properties in wound healing and regenerative medicine. In fact, it has been noted that snail mucus contains various bioactive compounds, such as glycoproteins, hyaluronic acid, enzymes and antimicrobial peptides. Below are summarized the main properties of this substance.

#### 5.2.1. Wound Healing

The human skin is composed of two layers: the outermost epidermis and the underlying dermis. It is the largest organ in the body. These layers consist of several types of cells, including dermal fibroblasts, keratinocytes, immune cells, nerves, and intradermal adipocytes [85]. Fibroblasts being located at the base of the collagen fibers are responsible for connecting the edges of wounds [28,86].

The process of wound healing is a multifaceted biological phenomenon that involves a sequence of events aimed at repairing damaged tissue and reinstating its structural and functional integrity through the interaction of various cellular components [87]. Following an injury to the skin, the sub-endothelium, collagen, and tissue factor are exposed, which triggers platelet aggregation. The process is governed by three overlapping phases: the haemostasis/inflammation phase, proliferation phase, and remodelling phase. Platelet degranulation releases chemokines and growth factors (GFs) to form a coat. Neutrophils are the first cells to appear at the wound area, Macrophages then accumulate and facilitate the phagocytosis of bacteria and damaged tissue [88].

It has been found that snail slime of *A. fulica* contains active ingredients, including heparan sulphate and calcium, which have been shown to have antibacterial and analgesic properties, as well as a role in haemostasis. In addition, several studies have demonstrated that heparan sulphate can accelerate wound healing by promoting blood clotting and cell proliferation. Furthermore, it has been discovered that snail mucus can encourage the growth of fibroblasts in the affected area, leading to a more rapid closure of wounds [89].

During the proliferation phase, Acharan sulfate, a glycosaminoglycan found in snail mucus, has been shown to form a complex important for wound healing [90]. This molecule was first discovered in 1996 by Kim et al. [87] through protease digestion of dried fat-free snail tissue. Acharan sulfate is a new type of 1→4-linked GAG, which is distinct from heparin or heparan sulfate. Acharan sulphate was chemically studied to define its anticoagulant activity. Its activity was evaluated by the Japanese group led by SJ Wu, using heparin as a standard and measuring clotting time and the conversion of chromogenic substrates with anti-factor IIa and Xa amidolytic [91]. In addition to these studies, a research group led by Prof Da-Wei Li evaluated the in vivo activity of the glycosaminoglycan Acharan sulphate. They found that its intravenous administration could prolong the clotting time measured by the aPTT and provide protection against thrombin-induced lethality [92].

In 2003, a study was conducted by the Faculty of Veterinary Medicine at the University of São Paulo to analyze the reparative activity of *A. fulica* mucus on skin lesions in rabbits. The animals were divided into three groups: pure mucus treatment, ointment treatment, and a no-treatment control group. The group treated with the mucus ointment showed the fastest healing, followed by the group treated with pure mucus, while the control group had a slower recovery. This study suggests that *A. fulica* mucus can accelerate wound healing in rabbits, especially when used as an ointment [93]. 

Another study was conducted on the use of films based on collagen and mucus secretion from *A. fulica* to treat wounds. The results showed that the treatment accelerated the formation and maturation of granular tissue and increased epithelialization. Moreover, there was a rapid replacement of type II collagen fibres with type I fibres on days 3 and 7, as observed with the microscope analysis. The study suggests that the treatments may have resulted in improved collagen fiber deposition and accelerated tissue regeneration after 14 and 21 days [94]. In addition, Sudjono T. et al., confirmed the therapeutic properties of *A. fulica* mucus using a gel formulation with carbomer 934 at concentrations of 3-7% and HPMC at concentrations of 6%, 8%, and 10%. The results of this experiment indicate that a higher concentration of gelling agents leads to increased viscosity and a longer healing effect. The snail mucus gel preparations containing 3% Carbomer 934 were found to be more effective in healing burns compared to the other preparations. The burn healing time was approximately 12.5±0.54 days, while the use of the gelling agent HPMC (6%) was more effective for burn healing for 12.67±0.33 days [95].

Additional medical applications have been investigated using an alginate membrane with a mixture of fulvic achatin mucus containing proteins, amino acids (e.g., proline, serine, asparagine, hydroxylin and threonine), and glycosaminoglycans to activate growth factors. The optimal composition of alginate/carboxymethl cellulose (CMC) and mucus was found to be in a 4:2 ratio, resulting in a dressing that effectively absorbed exudate and accelerated wound healing [96].

Recently some gelatin-chitosan-*Aloe vera* (AV)-*A. fulica* mucus scaffolds have been developed that thanks to their biocomposite, durability and porosity appearing to be potentially useful for tissue engineering in burns cases [97].

Professor Putri D. and colleagues performed a study demonstrating that *A. fulica* extract, which contains Acharan sulphate molecules, can increase the number of basal epithelial cells in a wound incision in the back of white male rats of Wistar strain by 86.74% in comparison with the control group [98]. Thus, the deposition of collagen is crucial for tissue repair, facilitated by the glycosaminoglycans present in the mucus. The concentration of snail mucus gel was found to affect collagen density and the rate of wound closure, as demonstrated in a 2021 experimental study by Putra A. et al. Significant differences in collagen density were observed between the control groups and the groups treated with 96% snail mucus gel [90].

The treatment of burn wounds has become a significant public health concern in recent years. It has been demonstrated that snail mucus has phytopharmacological potential to accelerate the inflammatory process during wound healing [99]. Tuo Deng and colleagues have been developing a natural biological adhesive with acharan sulphate from snail mucus. This adhesive matrix consists of positively charged proteins and polyanionic GAGs, which can adhere to wounded tissue. It has haemostatic, biocompatible, and biodegradable properties and is effective in accelerating wound healing. A study using a randomised experimental design, was conducted with 20 male mice divided into 5 treatment groups with different percentages of gels (3%, 4%, 5%). The parameters observed were burn healing time, burn diameter, and burn healing rate for 16 days. The results confirmed that burns healed with an average time of 14 days. The most rapid closure occurred at a concentration of 5%, with a healing rate of 98.35% [100].

#### 5.2.2. Antimicrobial Activity

Among the various beneficial activities of *Achatina fulica*, its antimicrobial property is particularly noteworthy. This characteristic has been identified in both mucus and haemolymph through the detection of several molecules. 

The scientific evidence of snail mucus was first discovered in 1982 by Dr Iguchi. It was demonstrated that the secretion obtained from the body surface of *A. fulica* inhibited the growth of both Gram-positive bacteria, *Bacillus subtilis* and *Staphylococcus aureus*, as well as Gram-negative bacteria, *E. coli* and *Pseudomonas aeruginosa* [101].

This effect was attributed to the protein component of the mucus, although a specific molecule was not identified initially. Later, Dr. Kubota isolated a glycoprotein with an approximate molecular weight of 160kDa, which exhibited highly positive antibacterial activity against the same strains used by Iguchi [102]. In 1992, another Japanese group led by Prof Otsuka-Fuchino officially named this glycoprotein Achacin [103]. Further studies elucidated the mechanisms employed by this molecule to inhibit bacterial growth, revealing its bacteriostatic effect by interacting with the cytoplasmic membrane and cell wall. This evidence was confirmed by a study conducted by Prof. Ehara in 2002, which demonstrated the L-amino acid oxidase activity of Achacin and its ability to generate cytotoxic H_2_O_2_ [104]. However, Achacin is not the only molecule isolated from *A. fulica*’s mucus. In 2011, a lectin designated as AfHML was purified, showing Ca^2+^-dependent hemagglutination activity. Despite this lectin didn’t inhibit bacterial growth, it induced agglutination of both gram-positive and gram-negative bacteria. [105] In fact, lectins are proteins that specifically bind to carbohydrates, showing great potential as therapeutic agents against bacterial infections by interfering with pathogen adhesion to host cells. A recent article explores the use of lectins in combined therapies for bacterial infections, positioning them as an alternative strategy to address antibiotic resistance. In particular, lectins, such as AfHML, could be a promising candidate due to their ability to bind to specific carbohydrates on bacterial surfaces. This suggests their potential role in preventing and treating conditions like acne vulgaris and other bacterial skin infections [105].

In subsequent years, the discovery of mytimacin-AF, a cysteine-rich antimicrobial peptide, added to the repertoire of antimicrobial molecules found in *A. fulica*. This peptide exhibited strong antimicrobial activity against a wide range of bacteria and fungi [106].

An innovative approach has been recently proposed using bioinformatics tools to predict bioactive peptides from the snail’s mucus of *Achatina fulica* that could have antimicrobial, anti-biofilm, cytotoxic, and cell-penetrating properties. Three promising peptides were selected, then modified to enhance their desired activities and then tested against *Propionibacterium acnes* isolates. The modified peptides showed strong antimicrobial activity against several strains, suggesting their potential for acne treatment and other medical uses [107].

Another recent study has highlighted the inhibitory effect of snail mucus against *Bacillus subtilis*, underscoring the ongoing exploration of *A. fulica* antimicrobial potential. In addition to antimicrobial activity, other effects such as antibiofilm and antiviral effects have also been studied [108,109]. For example, sulphated polysaccharides such as acharan sulphate, purified from the mucus, demonstrated the potential to inhibit the binding of the SARS-CoV-2 spike protein to the ACE2 receptor, suggesting broader applications beyond antimicrobial action [110].

Moving to haemolymph, it serves as a unique body fluid akin to human blood, albeit with crucial differences [111,112,113,114]. One of its components, C-reactive protein (ACRP), has shown remarkable abilities, including the sequestering of heavy metals and the stimulation of immune responses [115,116]. In addition, hemocyanin, the main component of haemolymph, plays a vital role in oxygen transport in invertebrates [117].

Moreover, lectins such as Achatinin H isolated from *A. fulica*’s haemolymph have been extensively studied [118]. These lectins exhibit calcium-dependent binding to specific sialoglyconjugates and demonstrate bacteriostatic effects against Gram-negative bacteria such as *E. coli*. Furthermore, studies on Achatinin’s involvement in the innate immune protection of *A. fulica* indicate its crucial role in defending against recurrent pathogen infections. Achatinin (H), a 9-O-Acetyl neuraminic acid specific lectin, was isolated from the hemolymph of the land snail *A. fulica* by affinity chromatography. The molecular weight of the native protein was 2.42 kDa [119]. UV-Vis absorption, fluorescence and circular dichroism spectroscopic studies on Achatinin (H) revealed the importance of divalent metal ions (Ca^2+^, Mg^2+^ and Mn^2+^) on lectin conformational change associated with its activity. [120,121]. Achatinin binds to specific sialoglyconjugates on lipopolysaccharide (LPS), exhibiting bacteriostatic effects on Gram-negative bacteria *E. coli* [122]. Achatinin participates in LPS-mediated coagulation of *A. fulica*’s haemolymph, indicating its crucial role in the innate immune protection of the snails [123].

It is evident that protection against recurrent pathogen infections is crucial for the biological invasion of *A. fulica*. The survival rate of giant African snails was recorded after a second infection with lethal doses of *E. coli*, following a previous injection using lipopolysaccharide (LPS). The log-rank test indicates that the survival rate of the LPS + Ec group was significantly higher than that of the other control groups after the second injection (*p* < 0.05). These results indicate that the giant African snail exhibits enhanced immune protection [124].

#### 5.2.3. Anticancer and Anti-Inflammatory Effect

Cancer, defined as an abnormal and uncontrolled growth of cells, represents a significant global health challenge [125]. It can metastasize to other tissues and organs, often leading to severe health complications and death [126]. Cancer can affect virtually any part of the body and can arise from various factors, including genetic mutations, environmental exposures, lifestyle choices, and infectious agents [127]. It is a leading cause of morbidity and mortality worldwide, posing significant challenges to healthcare systems and societies at large [128]. Despite advancements in research and treatment, cancer remains a formidable global health burden, underscoring the ongoing need for prevention, early detection, and innovative therapeutic strategies [129]. For these reasons in the last decades different sources have been proposed for the development of new anticancer agents. One of these was *Achatina fulica*. In this section all the scientific studies published on this topic are summarized. 

In 1987 in the haemolymph of this snail species a sialic acid-binding lectin called Achatinin H with highly specificity for 9-O-acetyl sialic acid was discovered [130]. Further chemical analyses revealed it is a galactose specific agglutinin, with a native molecular weight of 210kDa and composed of non-covalently linked identical subunits of molecular weight 15kDa. Usually, Achatinin H does not agglutinate normal human erythrocytes, but it is able to agglutinate erythrocytes from patients with acute lymphoblastic leukaemia and acute myeloid leukemia. Surprisingly, the same dose of Achatinin H that caused mitogenic activity in resting lymphocyte culture inhibited PHA-induced blastogenesis [131]. But considering that elevated levels of anti-9-O-acetylated sialoglycoconjugates (9-OAcSGs) were identified in paediatric acute lymphoblastic leukaemia (ALL) patients an ELISA analysis was developed to monitor serum levels of anti-9-OAcSGs as potential markers for ALL diagnosis and monitoring [132]. So, from the exploration of the selective affinity of Achatinin (H) towards terminal 9-O-acetylated sialic acids-α2-6-Nacetylated galactosamine has been observed enhanced expression of OAcSGP at the onset of disease, followed by its decrease with chemotherapy and reappearance with relapse [117]. Current research into natural products like anticancer therapies is promising. In fact, a study has demonstrated that a dose of 25 mg/kg BW of the whole snail mucus of *A. fulica* has inhibitory activity on mammary cancer growth in Sprague-Dawley rats induced with 7,12-dimethylbenz (α)anthracene (DMBA) for five weeks. In addition, the histopathology results of the snail mucus showed normal tissue depiction and histopathological form [133]. Also, Achatinin (H), exhibited cytotoxic effects on the MCF7 human mammary carcinoma cell line. In fact, morphological changes resulting in cell death, cell cycle arrest, and apoptosis induction were observed in this kind of cells treated with Achatinin H [134]. 

Another extremely interesting molecule was discovered by Kim and colleagues in 1996 in *A. fulica* mucus, called Acharan sulfate. Then it has been extensively studied elucidating its chemical composition [135]. It is a glycosaminoglycan with a primary repeating disaccharide structure of alpha-D-N-acetylglucosaminyl-2-O-sulfo-alpha-L-iduronic acid [136]. Its beneficial properties have been also explored. For example, the antiangiogenic activity of Acharan sulfate was evaluated through the chorioallantoic membrane assay and by measuring its effect on the proliferation of calf pulmonary artery endothelial cells. In vivo, a matrigel plug assay demonstrated that Acharan sulfate inhibited basic fibroblast growth factor (bFGF)-stimulated angiogenesis and reduced the hemoglobin (Hb) content within the plug. In addition, intraperitoneal injection of Acharan sulfate resulted in a 40% decrease in tumor weight and volume in sarcoma 180-bearing mice [137]. The inhibitory effect of Acharan sulphate on angiogenesis has been studied also by other research groups observing that in carrageenan- and cotton thread-induced granulation tissues it is not due to the inhibition of VEGF protein induction but due to the inhibition of VEGF-induced vascular tube formation [138]. Acharan sulfate has also been studied for its role in regulating physiological processes through interactions with a diverse range of proteins. Subcutaneous injection of exogenous Acharan sulfate was administered near the tumor tissue in C57BL/6 mice that had been implanted with Lewis lung carcinoma cells (LLCs). The results indicate that Acharan sulfate may inhibit tumor growth by binding to the nucleolin receptor protein on the surface of cancer cells. In fact, this molecule of 110kDa, when phosphorylated, acts as a cell surface receptor for various ligands, such as growth factors and chemokines [139]. Further investigations have also revealed that Acharan sulfate is cytotoxic to cancer cells and strongly interacts with specific cell-surface proteins playing a critical role in inhibiting tumors [140]. 

However, it is not only the mucus that has beneficial effects on anti-inflammatory responses. In fact, it has also been demonstrated that Haemocyanin, which is known for its anti-trypsin activity, inhibits the release of histamine, 5-hydroxytryptamine and possibly prostaglandins E1 and E2 [141]. Heparan sulphate and acharan sulfate in snail mucus are known for their anti-inflammatory properties. In a study that evaluated the impact of snail mucus on the activity and chronicity indices of renal histology in a pristane-induced lupus nephritis mice model, snail mucus significantly decreased the activity index of renal histology, indicating its potential in mitigating renal inflammation in lupus nephritis [142].

In recent years, researchers have successfully synthesized silver nanoparticles from the snail *A. fulica* using a clean and easily scalable method. These biogenically synthesized silver nanoparticles demonstrated even anticancer activity, inhibiting Hela cells by more than 15%. This suggests the potential for formulating antimicrobial and potentially anticancer creams or gels for topical application in skin ailments. It is important to note that further research is needed to fully understand the potential benefits and risks of this approach [143].

Finally, it should be noted bioinformatic analysis as an exceptional tool for biological researchers. For example, It resulted in being able to predict potential anticancer peptides from *A. fulica* mucus fractions, indicating their potential for drug development [144]. 

#### 5.2.4. Osteoinduction Activity and Dental Applications

The potential therapeutic benefits of *A. fulica* mucus in dental diseases and bone regeneration are significant, offering promising applications in oral health and regenerative medicine. Dental and bone-related disorders pose considerable challenges to global public health. The bioactive compounds found in *A. fulica* secretion exhibit properties that may be useful in treating these conditions. *A. fulica* mucus contains antimicrobial peptides that effectively inhibit the growth of oral pathogens including *Streptococcus mutans and Porphyromonas gingivalis* [145,146]. This can help prevent dental caries and periodontal infections. In addition, the anti-inflammatory properties of *A. fulica* mucus can alleviate gingival inflammation and promote periodontal tissue regeneration, further supporting oral health maintenance and recovery from oral diseases. This mucus could facilitate osteogenic differentiation of mesenchymal stem cells and enhance mineralization. In fact, periodontal disease causes bone damage by altering osteoclast and osteoblast activity in response to local inflammation, which is aided by the presence of glycosaminoglycans. Studies have shown that *A. fulica* mucus can decrease the number of osteoclasts, potentially aiding in the healing process in periodontitis [147]. A study led by Handrawati F. determined and proved the effect in increasing the number of osteoblasts in rat models suffering from periodontitis [148].

Other studies have also observed the mineralization and gene expression in dental pulp cells following treatment with *A. fulica* mucus [149]. The results demonstrated increased mineralized nodules and expression of osteopontin and NF-κB, indicating the differentiation of dental pulp cells into bone cells. Furthermore, *A. fulica* mucus induced the osteogenic differentiation of human mesenchymal stem cells and human fetal osteoblastic cell lines, as evidenced by upregulated expression of osteogenic markers such as osteopontin and osteocalcin [150].

The regenerative potential of *A. fulica* mucus extends to bone tissue, accelerating bone healing processes and improving bone density through modulation of key signaling pathways such as BMP-2 and Wnt/β-catenin. Furthermore, its immunomodulatory effects aid in reducing inflammation at the site of bone injury, creating an optimal environment for regeneration [151]. Despite this promising evidence, further research is required to fully explain the mechanisms of action and optimize the clinical applications of this treatment in oral health and regenerative medicine. 

## 6. Conclusions and Future Perspectives

*Achatina fulica*, more commonly known as the African giant land snail, stands as a prominent example of an invasive species wreaking havoc on ecosystems and public health worldwide. Its extensive distribution and role as an intermediate host for nematodes, including those linked to eosinophilic meningitis, underscore its detrimental impact. However, recent scientific investigations have unveiled a fascinating array of benefits associated with Achatina and its remarkable mucus.

Explorations into *A. fulica* mucus have unveiled a treasure trove of therapeutic potential. Studies have illuminated the presence of antimicrobial, anti-inflammatory, and antioxidant properties, unlocking promising avenues for medical applications such as wound healing and treatment of bone and dental diseases.

The paradox of *A. fulica* lies in its dual nature, it is both a menace and a marvel. Yet, understanding its ecological and socio-economic dimensions is paramount in charting a path forward. In affluent nations, robust financial resources can fuel eradication endeavours through stringent regulations and enforcement mechanisms, aiming to curb its spread and safeguard ecosystems and public health.

Conversely, in economically disadvantaged regions, innovative approaches are imperative. Here, the presence of *A. fulica* can catalyse the adoption of circular economy practices. By repurposing these snails for endeavours like animal feed or fertilizer, communities can transform a nuisance into a valuable economic asset. This not only bolsters local economies but also fosters sustainability by minimizing waste and maximizing resource efficiency.

The versatility of *A. fulica* extends beyond its role in ecosystems and public health. It permeates various industries, from agriculture to biofuel production, serving as a sustainable source of animal feed and a raw material for biodiesel and nanoparticle synthesis. Its utilisation underscores the potential for harmonizing economic prosperity with environmental stewardship.

As global interest in *A. fulica* surges, it is imperative to tread cautiously, navigating the delicate balance between economic viability and environmental preservation. Effective regulatory frameworks and ecological monitoring are indispensable tools in this endeavour, ensuring that its invasive tendencies are mitigated while its beneficial contributions are harnessed responsibly.

In essence, *A. fulica* embodies a nuanced dichotomy, embodying both a challenge and an opportunity. By harnessing its positive attributes judiciously, we can envision a future where it coexists harmoniously with human well-being and economic prosperity, all while minimizing its ecological footprint.

## 7. Patents

The following table provides a comprehensive overview of patents related to various applications of substances derived from the giant African snail (*Achatina fulica*) and other biological sources. These patents cover a wide range of fields, including biotechnology, medicine, agriculture and cosmetics. Each entry specifies the patent title, the country where the patent was filed and the corresponding application number. This compilation highlights the diverse and innovative uses of natural compounds in scientific and commercial developments (Table 1).

## Figures and Tables

**Table 1:** List of patents related to biotechnological, medical, agricultural and cosmetic applications based on substances derived from the giant African snail (*Achatina fulica*) and other organisms. The table includes the patent title, the country of registration and the associated application number.

Argument	Country Office	Application Number
Isolation, Purification and Characterization of Heparin-Like Substances	Thailand; China; Usa; European Patent Office	21917982; Wo/2022/150018; 202180085309.3 18265655
*Achatina fulica* extract	Republic Of Korea	1019950046690;
Breeding pond for *Achatina fulica*	China	201710304064.4
Production method of quick-frozen seasoned *Achatina fulica*	China	201810789066.1
Preparation method of Babylonia (gastropod) aquaculture feed by *Achatina fulica*	China	201610199543.X
Heparosan-Glucuronic Acid-5-Epimerase, and method for producing polysaccharide using same	European Patent Office; Usa	14811007 Wo/2014/200045 14897667
Osseo-integrator comprising of porous chitosan and spermatheca gland extract of snail *Achatina fulica* for effective hard tissue repair	India	192/Kol/2012
D-Type amino acid-containing neuropeptide	Japan	1995177870
Empty shell of giant African snail (*Achatina fulica*) as lighting lamp (Diya)	India	202031024498
Producing natural feeds for freshwater catfish	Philippines	2/2018/000763
Protein mucus of *Achatina fulica* Ferussac (Achasin) Javanese strain as an antibacterial liquid	Indonesia	P00201702661
Fertilizer for improving yield of *Shatian pomelo*; fertilizer capable of effectively enhancing sugarcane yield; a fertilizer increasing the yield of illicium verum; fertilizer for promoting growth of fast-growing eucalyptus	China	201611057191.0; 102016001057198 102016001057184 102016001057201
Fulicin-like neuropeptide	Japan	1994061928
Use of acharan sulfate for treatment and prevention of hyperlipidemia	Republic of Korea	1020010018148
Snail enzyme preparation method; preparation method of snail protease	China	201510188175.4 201510188191.3
Compact nourishing lyophilized powder facial mask	China	102017000331765
Life-nourishing health-care noodles capable of loosening bowels and clearing away toxin, and preparation method of noodles	China	201610680855.2
Process for producing canned edible snail (*Achatina fulica,* escargots)	Republic of Korea	1020000000542
African snail attractive toxicant based on invasive plants and snail pheromones, and preparation method of African snail attractive toxicant	China	202110707216.1
Medicine for treating blood-insufficiency and yin-deficiency recurrent oral ulceration and preparation method thereof	China	201510167973.9
Cosmetic composition containing mucus of snails which are fed with red ginseng and a method for preparing the same	Republic Of Korea	1020110133374
Making method of spicy sauce	China	201910000098.3
An improved process for the isolation of a new, highly specific 9-0-acetylated sailoglycoconjugate-binding lectin (Achatinin-h) from *Achatina fulica* snail, useful for the diagnosis of visceral leishmaniasis.	India	2502/Del/1996

## Data Availability

This section is not applicable to this specific literature review.

## Supplementary Materials

The following supporting information can be downloaded at: https://www.mdpi.com/article/10.3390/biology14020190/s1, Table S1: Historical records of Achatina fulica introduction worldwide, from the early 19th century to the present. The table lists the year of first recorded occurrence, the country of introduction, and the corresponding reference [1,7,31,41,80,152,153,154,155,156,157,158,159,160,161,162,163,164,165,166,167,168,169,170,171,172,173].

## Abbreviations

9-OAcSGs	9-O-acetylated sialoglycoconjugates
A. abstrutus	Angyostrongylus abstrutus
A. costaricensis	Angyostrongylus costaricensis
A. vasorum	Angyostrongylus vasorum
A.cantonensis	Angyostrongylus cantonensis
A.fulica	*Achatina fulica*
ACRP	C-reactive protein
AfHML	Achatina fulica High Molecular Lectin
Ag-NP	Silver nanoparticles
ALL	Acute lymphoblastic leukaemia
aPTT	Activated partial thromboplastine time
AV	Aloe vera
bFGF	Basic fibroblast growth factor (bFGF)
BMP-2	Bone morphogenetic protein 2
BW	Body weight
C. frutescens	Capsicum frutescens
DMBA	7,12-dimethylbenz (α)anthracene
E. rosea	Euglandina rosea
GAG	Glycosaminoglycans
GPs	Growth factors
Hb	Hemoglobin
HPMC	Hydroxypropyl methylcellulose
LAB	Lactic acid bacteria
LLCs	Lewis lung carcinoma cells
LPS	Lipopolysaccharides
mitamycin-AF	Mitamycin Achatina fulica
MRD	Minimal residual disease
NF-κB	Nuclear factor kappa-light-chain-enhancer of activated B cells
P. hermaphrodita	Phasmarhabditis hermaphrodita
PAS	Periodic acid-Schiff reagent
R.rattus	Rattus rattus
SSCA	Autoclave-deacetylated snail shell chitosan
SSCU	Ultrasound-deacetylated snail shell chitosan
VEGF	Vascular endothelial growth factor
WOS	Web of Science
