# Supplementary material for: Evaluation of Biological Properties and Beneficial Effects for a Sustainable and Conscious Exploitation of Achatina fulica Snails
Source: Biology (Basel). 2025 Feb 12;14(2):190. doi: 10.3390/biology14020190 (PMC11851829; doi:10.3390/biology14020190)
Supplement: Supplementary file 1 [file biology-14-00190-s001.zip › biology-3406933-supplementary.pdf]

Table S1. Historical records of *Achatina fulica* introduction worldwide, from the early 19th century to the present. The table lists the year of first recorded occurrence, the country of introduction, and the corresponding reference.

Year	Country	Reference
1822	East Africa	[152]
1847	India	[7]
19th century	Ghana	[153]
19th century	Madagascar	[154,155]
19th century	Camoros	[31]
19th century	Mayotte	[31]
19th century	Ivory Coast	[31]
1900	Sri Lanka	[154]
1911	Malaysia	[154]
>1917	Singapore	[154]
1928	Borneo	[154]
1930	Ogasawara and Chichijima Island Japan	[154,156]
1931	Philippines	[157]
<1937	Thailand	[154]
<1937	Vietnam	[154]
1937	Hong Kong	[154]
1941-43	Guam	[158]
1942	Indonesia	[159]
1943	Santhal Parganas region	[160]
<1945	New Guinea	[159]
1945-50	New Ireland	[158]
1951	Bengal, Orisaa, Kerala and Andam-Nicobar Island	[160]
<1957	Maldive	[161]
<1960	Bangladesh	[154,162]
1966	Florida	[163]
1967	Taiwan	[164]
<1968	Cambodia	[165]
1969	French Polynesia	[157]

<1972	Bali	[163]
1972	New Caledonia	[158,163]
1980	Guadeloupe and Martinique	[41]
1980	Brazil	[166]
1983	Morocco	[31]
1984	Guadalupe	[1]
1987	Mauritus	[167]
2000	St. Lucia and Barbados	[41]
2005	Ecuador	[161,168]
2007	Argentina	[166]
2007	Paraguay	[166]
2007	Chile	[169]
2007	Uruguay	[169]
2007	Bolivia	[80]
2008	Trinidad	[170]
2008	Antigua	[171]
2008	Colombia	[172]
2012-13	Dapeng Peninsula of Shenzhen City (China)	[173]
